# Thromboembolic events and apparent heparin resistance in patients infected with SARS‐CoV‐2

**DOI:** 10.1111/ijlh.13230

**Published:** 2020-06-15

**Authors:** Robert Beun, Nuray Kusadasi, Maaike Sikma, Jan Westerink, Albert Huisman

**Affiliations:** ^1^ Department of Intensive Care University Medical Center Utrecht and University Utrecht Utrecht the Netherlands; ^2^ Dutch Poisons Information Center University Medical Center Utrecht and University Utrecht Utrecht the Netherlands; ^3^ Department of Vascular Medicine University Medical Center Utrecht and University Utrecht Utrecht the Netherlands; ^4^ Department of Clinical Chemistry and Laboratory Medicine University Medical Center Utrecht and University Utrecht Utrecht the Netherlands

Dear Editors,

In March 2020, the global healthcare system is overwhelmed by patients infected with SARS‐CoV‐2, which is the cause of the coronavirus pandemic (Covid‐2019). A large number of these patients end up in the intensive care units with critical illness requiring mechanical ventilation. One of the most important clinical features of the infection is a profound coagulopathy. In a recent cohort study, 71% of patients who eventually died matched the ISTH criteria for disseminated intravascular coagulation, while this percentage was only 0.6% in patients who survived.^1^ Although no data have been presented or published, clinical experience suggests that this coagulopathy is associated with an increased risk of both venous and arterial thrombosis. Treatment of thrombosis in the setting of profound coagulopathy may be hampered by the inability to use the aPTT ratio when treating with unfractionated heparin (UHF). We provide data from our center and provide guidance for treatment of perceived heparin resistance associated with the coagulopathy in patients with SARS‐CoV‐2 infection.

In our hospital, 75 patients have now been admitted to the ICU with SARS‐CoV‐2 infection, some of these experiencing serious thromboembolic complications during ICU stay including pulmonary embolism, ischemic‐CVA, acral ischemia, and recurrent clotting of dialysis filters or oxygenators of extracorporeal membrane oxygenation. The percentage of venous thromboembolic events diagnosed at the time of suspicion can be seen in Table 1.

**TABLE 1:** Thromboembolic events in patients with SARS‐CoV‐2 pneumonia

	Number of patients (% of total)
Total number of patients admitted to ICU	75
Clinical suspicion of thromboembolic event	35 (46.6%)
Performed diagnostic approach	CT: 32 and ultrasound: 3
Pulmonary embolism in (sub)segmental arteries	16 (21.3%)
Pulmonary embolism in central artery	4 (5.3%)
Ischemic cerebrovascular accident	2 (2.7%)
Deep vein thrombosis	3 (4.0%)
Patients with thromboembolic event (total)	25 (33.3%)

Note

Patients admitted to the ICU from March 16 until April 9.

These patients were therapeutically treated with either UHF intravenously (UFH) (with an aPTT ratio range between 2.0 and 3.0 (ie, 50‐75 seconds)) or low–molecular‐weight heparin. Table 2 provides data on four patients with an indication for therapeutic anticoagulation and profound coagulopathy at admission or diagnosis of a thromboembolic event. Treatment in all four patients was complicated by necessitating very high UFH doses to achieve perceived adequate coagulation, based on the aPTT ratio.

**TABLE 2:** Results of Factor VIII, fibrinogen, D‐dimer, antithrombin, and the platelet count

	Mean (Min.–Max.)	Reference range
Age (years)	60.5 (53‐68)	n.a.
Gender (M/F)	2/2	n.a.
UFH (Max. IU/24 h)	48 708 (36 748‐64 576)	n.a.
Factor VIII (IU/mL)	4.45 (2.50‐5.89)	0.60‐1.50
Fibrinogen (g/L)	7.3 (6.9‐7.6)	2.0‐4.0
D‐dimer (mg/L)	48.6 (13.8‐100)	<0.5
Antithrombin (IU/mL)	0.91 (0.67‐1.19)	0.80‐1.20
Platelet count (×10^9^/L)	270 (223‐302)	150‐450

Note

Blood samples were drawn on the day of admission to the ICU, or on the first day of signs of thromboembolic events. Conversion factor for Factor VIII and antithrombin: 1.00 IU/mL = 100 IU/dL = 100%.

Abbreviation: n.a.: not applicable.

This rare phenomenon defined as the need for high‐dose UFH of more than 35 000 IU/d to achieve the target aPTT ratio or the impossibility of doing so is called heparin resistance. In an attempt to elucidate the possible causal factor(s) of heparin resistance in patients with a SARS‐CoV‐2 infection, we measured the levels of the coagulation‐associated factors such as Factor VIII, D‐dimer, fibrinogen, and antithrombin in these patients. Coagulation assays were measured in the central ISO‐15189 accredited laboratory using an ACL Top 750 LAS coagulation analyzer (Werfen Diagnostics) using Coamatic Factor VIII reagent (chromogenic Factor VIII), QFA thrombin reagent (fibrinogen), liquid antithrombin reagent (antithrombin), and D‐dimer HS500 reagent (D‐dimer) all obtained from Werfen diagnostics. Platelets were measured using an Alinity Hq hematology analyzer (Abbott Diagnostics). A formal ethics review for this study was deemed unnecessary by the local ethics committee.

Factor VIII was found to be extremely increased in SARS‐CoV‐2 patients, also fibrinogen and d‐dimer were elevated, while almost all of the antithrombin levels were in the normal range. High Factor VIII level is a common cause of apparent heparin resistance.^2^, ^3^ Increased Factor VIII levels decrease or normalize the in vitro anticoagulant activity of heparin as measured by aPTT, while the in vivo antithrombotic activity of heparin remains unaffected as measured by anti‐Xa assay.^4^ Patients requiring high‐dose UFH to achieve the target aPTT without monitoring its antithrombotic activity via an anti‐Xa assay may develop life‐threatening bleeding complications. Previous studies have shown the anti‐Xa level as a more suitable parameter for monitoring the antithrombotic activity than the aPTT.^5^, ^6^, ^7^ Monitoring the anti‐Xa level shortens the time to reach the target therapeutic range as well as improves the length of time in the target range.^5^, ^6^, ^7^ The treatment and monitoring complications observed with the use of UFH necessitate us to adjust the clinical guidelines used locally awaiting (inter)national guidance.

Based on the limited SARS‐CoV‐2 data and our own experience, we suggest to monitor the heparin activity of UFH treatment based on anti‐Xa levels with a target value of 0.3‐0.7 U/L in all patients with SARS‐CoV‐2 instead of treatment based on aPTT levels.^5^, ^6^, ^7^

## CONFLICT OF INTEREST

None.
